# Bilberry extract (Antho 50) selectively induces redox-sensitive caspase 3-related apoptosis in chronic lymphocytic leukemia cells by targeting the Bcl-2/Bad pathway

**DOI:** 10.1038/srep08996

**Published:** 2015-03-11

**Authors:** Mahmoud Alhosin, Antonio J. León-González, Israa Dandache, Agnès Lelay, Sherzad K. Rashid, Claire Kevers, Joël Pincemail, Luc-Matthieu Fornecker, Laurent Mauvieux, Raoul Herbrecht, Valérie B. Schini-Kerth

**Affiliations:** 1 CNRS UMR 7213 Laboratoire de Biophotonique et Pharmacologie, Université de Strasbourg, Faculté de Pharmacie, 74, route du Rhin, 67401 Illkirch, France; 2 Oncologie et Hématologie, Hôpitaux Universitaires de Strasbourg, Avenue Molière, 67100 Strasbourg, France; 3 University of Liège, Plant Molecular Biology and Biotechnology Unit, B22, Sart Tilman, B-4000 Liège, Belgium; 4 Dept. of Cardiovascular Surgery and CREDEC, Pathology Tower B23, Sart Tilman, B-4000 Liège, Belgium; 5 Université de Strasbourg, Faculté de Médecine, Strasbourg, France; 6 Laboratoire d'Hématologie, Hôpitaux Universitaires de Strasbourg, Avenue Molière, 67100 Strasbourg, France

## Abstract

Defect in apoptosis has been implicated as a major cause of resistance to chemotherapy observed in B cell chronic lymphocytic leukaemia (B CLL). This study evaluated the pro-apoptotic effect of an anthocyanin-rich dietary bilberry extract (Antho 50) on B CLL cells from 30 patients and on peripheral blood mononuclear cells (PBMCs) from healthy subjects, and determined the underlying mechanism. Antho 50 induced concentration- and time-dependent pro-apoptotic effects in B CLL cells but little or no effect in PBMCs. Among the main phenolic compounds of the bilberry extract, delphinidin-3-*O*-glucoside and delphinidin-3-*O*-rutinoside induced a pro-apoptotic effect. Antho 50-induced apoptosis is associated with activation of caspase 3, down-regulation of UHRF1, a rapid dephosphorylation of Akt and Bad, and down-regulation of Bcl-2. Antho 50 significantly induced PEG-catalase-sensitive formation of reactive oxygen species in B CLL cells. PEG-catalase prevented the Antho 50-induced induction of apoptosis and related signaling. The present findings indicate that Antho 50 exhibits strong pro-apoptotic activity through redox-sensitive caspase 3 activation-related mechanism in B CLL cells involving dysregulation of the Bad/Bcl-2 pathway. This activity of Antho 50 involves the glucoside and rutinoside derivatives of delphinidin. They further suggest that Antho 50 has chemotherapeutic potential by targeting selectively B CLL cells.

Chronic lymphocytic leukemia (CLL), defined by the accumulation of pathogenic B cells, is still an incurable disease despite the recent development of novel therapeutic approaches. Dysregulation of apoptosis is a hallmark of CLL making this disease an ideal experimental model of malignancy due to the failure of apoptosis^1^. The current knowledge about the mechanism responsible for dysregulation of apoptosis in CLL is still poor. High levels of the anti-apoptotic protein Bcl-2 have been found in several hematological malignancies, including CLL^2^,^3^. Therefore, overexpression of Bcl-2 has been suggested to have a central role in defects of apoptosis and cell survival observed in CLL^4^. The opportunity to induce apoptosis by targeting Bcl-2 protein and/or Bcl-2-regulating proteins such as Bad is therefore considered as a promising therapeutic approach in CLL^5^. Several epidemiological studies have suggested that the consumption of fruits and vegetables can reduce the risk of developing cancer. Polyphenol-rich products have been reported to have potential chemopreventive and chemotherapeutic activities in cancer cells including CLL by targeting several apoptosis-regulating pathways^6^,^7^. In this context, green tea and its active constituent epigallocatechin gallate (EGCG) have been shown to induce apoptosis in leukemic B cells isolated from CLL patients^8^. At low concentrations, EGCG significantly increased apoptosis in CLL cells involving Bcl-2 down-regulation, caspase 3 activation and the dephosphorylation of VEGF receptors^8^. Inhibition of the endogenous nitric oxide pathway causing caspase 3 activation during apoptosis has also been suggested to contribute to the pro-apoptotic effects of polyphenolic compounds such as derivatives of resveratrol and viniferin^9^.

Beneficial effects of berries have been reported in several diseases such as cardiovascular diseases and cancer^10^,^11^. The protective and anti-cancer effects of berries have been predominantly attributed to their high content of polyphenolic compounds, especially anthocyanins^10^,^11^. Bilberry (*Vaccinium myrtillus* L.) is one of the richest dietary natural sources of anthocyanins which has been shown to have several therapeutic activities such as inhibition of angiogenesis and anti-cancer properties^10^,^12^. Antho 50 is a bilberry extract composed of about 50% of anthocyanins which has previously been shown to be predominantly absorbed as glycosides from the stomach in rats^13^. The aim of the present study was to evaluate the pro-apoptotic effects of this bilberry extract rich in anthocyanins on CLL cells from 30 patients and peripheral blood mononuclear cells (PBMCs) from 5 healthy subjects and, if so, to determine the signaling pathway involved.

## Methods

### Patients, cell separation, and culture conditions

All experiments were performed in accordance with the Declaration of Helsinki and approved local ethical guidelines. Patients received oral and written information on research and all signed a consent form approved by the Ethic Committee (Comité de Protection des Personnes “Est-IV”, 1 place de l'Hôpital, 67091 Strasbourg Cedex, France). Cells were collected from 30 patients (21 male, 9 female) at the University Hospital of Strasbourg, France (Table 1). Median age of the patients was 69 years (range: 43–83 years). Median circulating lymphocytes count was 53.3 × 10^3^/μL (range 4.2–190.2 × 10^3^/μL). Twenty-three patients were untreated for CLL while 7 had received 1 to 4 prior lines of chemotherapy. All these 7 patients were off-therapy for at least two months at time of cells sampling. Five peripheral blood samples have been sampled from donors and used in the study. Disease has been characterized in all patients by increased lymphocyte count in blood, typical cytological aspects of the cells and immunophenotyping showing a monotypic cell population with a Matutes score of 4 or 5. Peripheral blood mononuclear cells (PBMC) were isolated by Ficoll density-gradient centrifugation (Lymphocyte Separation Medium, MP Biomedicals). Cells were incubated at 1 to 2 × 10^6^ cells/mL in RPMI 1640 medium containing 10% fetal bovine serum and incubated at 37°C in an atmosphere of 5% CO_2_.

### UPLC-PDA analysis

Bilberry anthocyanin purified extract, Antho 50, was kindly provided by FERLUX S. A. (Cournon d'Auvergne, France). Standards of cyanidin-3*-O-*gluoside, cyanidin-3*-O-*galactoside, cyanidin-3*-O-*rutinoside, delphinidin-3*-O-*gluoside, delphinidin-3*-O-*galactoside and delphinidin-3*-O-*rutinoside were purchased from Extrasynthese (Genay Cedex, France).

Antho 50 was hydrolyzed in acidic conditions as previously described^14^.

UPLC-PDA analysis of Antho 50 and the hydrolyzed extract was performed in a liquid Acquity chromatograph (Waters) equipped with a photodiode array detector (PDA). Separation was carried out at 50°C using an acquity BEH C18 column (Merck), 100 mm × 2.1 mm, filled with 1.7 μm particles. The elution gradient was performed using water, acetonitrile and formic acid as solution: a linear gradient from 96% water to 75% in 10 min followed by a linear gradient from 75% to 96% water in 2 min; always with 2% formic acid. Flow rate was 0.2 mL/min. Absorbance was recorded at 518 nm.

### Measurement of total phenolic compounds and total anthocyanins

Total phenolic content was determined according to the Folin-Ciocalteu method as previously described^15^. The results were expressed in mg gallic acid equivalents (GAE) per gram of extract. Total polyphenolic content was 513.20 ± 16.20 mg GAE/g.

The determination of anthocyanin index was performed by the Ribereau-Gayon method according to González-Rodríguez et al^16^. The results were expressed in mg anthocyanins per gram of extract. The total anthocyanin content was of 450.31 ± 5.70 mg/g.

### Apoptosis analysis

The annexin V-FITC/PI apoptosis assay (BD Biosciences Pharmingen, San Diego, CA, USA) was used to detect apoptosis. Experiments were performed according to the manufacturer's instructions on PBMCs and B cells from CLL patients exposed to Antho 50 or to a pure anthocyanin at different concentrations and for different times. Apoptosis rates were then assessed by flow cytometry. At least 10,000 events were recorded and represented as dot plots.

### Cell viability assay

Cells were seeded on 6-well plates at a density of 2 × 10^6^ cells/well, grown for 24 h and exposed to Antho 50 at 75 μg/mL for different times. Cell viability ratio was determined by cell counting using the trypan blue exclusion method (Invitrogen). The viability rate was obtained by dividing the number of trypan blue-negative cells (viable cells) by the total number of cells.

### Assessment of DNA fragmentation pattern

Genomic DNA was prepared according to the manufacturer's instructions (Qiagen, Courtaboeuf, France), separated by electrophoresis on a 1% agarose gel and visualized under UV light with ethidium bromide.

### Western blot analysis

Cells were grown in 6-well plates and treated with different concentrations of Antho 50 for different times. Cells were lysed with ice-cold RIPA buffer (150 mM NaCl, 1% Triton X-100, 0.5% Na deoxycholate, 0.1% SDS, 50 mM Tris-HCl, pH 7.5, and a protease inhibitor mixture tablet). Equal amounts of total proteins were separated on 10–12% polyacrylamide gel and electrophoretically transferred to nitrocellulose membranes (GE Healthcare, Buckinghamshire, UK), which were then blocked with 5% BSA (BioRad, Hercules, USA) for 1h30 at room temperature. Membranes were then incubated with either a mouse monoclonal anti-UHRF1 (Proteogenix, Oberhausbergen, France), a rabbit polyclonal anti-caspase 3, a rabbit polyclonal anti-p-Bad, a rabbit polyclonal anti p-Akt Ser473 (Cell Signaling Technology, Inc. Danvers, MA), a mouse monoclonal anti-p73 (BD Biosciences Pharmingen), a mouse monoclonal anti-p53 (Santa Cruz Biotechnologies, Santa Cruz, CA, USA), a mouse monoclonal anti-Bcl-2 (Millipore, Darmstadt, Germany) or a mouse monoclonal anti-β-tubulin or anti-β-actin antibody (Abcam, Paris, France), according to the manufacturer's instructions at 4°C overnight. The membranes were then washed three times; 5 min/each time with PBS. Membranes were thereafter incubated with the appropriate horseradish peroxidase-conjugated secondary antibody (diluted to 1:10,000 for anti-mouse antibody and 1:5,000 for anti-rabbit antibody) at room temperature for 1 h. Membranes were then washed with PBS five times. Signals were detected by chemiluminescence using an enhanced chemiluminescence kit (GE Healthcare).

### Determination of the cellular formation of reactive oxygen species

The oxidative fluorescent dye dihydroethidium (DHE) was used to evaluate the formation of reactive oxygen species (ROS). To determine the nature of ROS, cells were incubated either with superoxide dismutase (SOD, 500 U/mL), catalase (500 U/mL), or PEG-catalase (membrane permeant analog of catalase, 500 U/mL) for 30 min at 37°C. Then, cells were challenged with or without Antho 50 (75 μg/mL) for different times followed by the addition of DHE (5 μM) for 15 min. After staining with DHE, cells were subjected to flow cytometry examination (BD FACSCalibur, Becton Dickinson, Franklin Lakes, NJ, USA). Histograms of 10,000 events were recorded per experiment.

### Statistical Analysis

Results are presented as mean ± SEM of at least three independent experiments. In the case of pairwise between group comparisons, statistical analysis was carried out using Student's *t* test. Statistical analysis was also performed using a two-way analysis of variance (ANOVA) followed by a Bonferroni post-hoc test to compare differences. Significant differences are indicated as *P < 0.05, **P < 0.001, ***P < 0.0001.

## Results

### Antho 50 selectively induces apoptosis in B CLL cells

**Figure 1:**
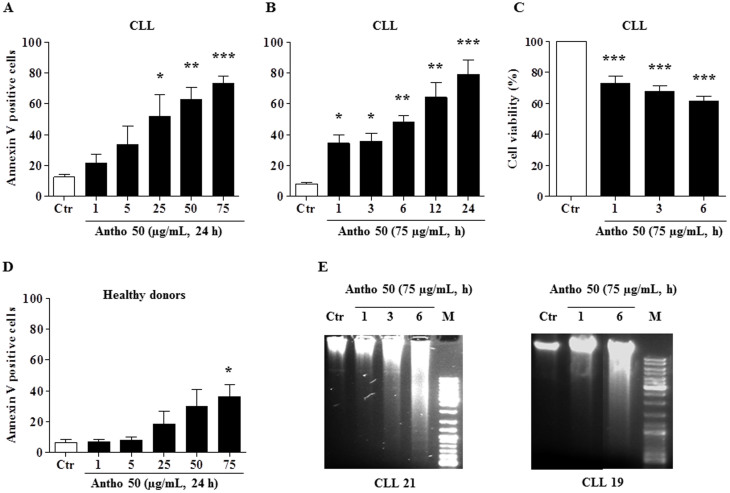
Antho 50 reduces cell viability and induces selectively a concentration- and time-dependent apoptosis in B CLL cells. Cells were exposed to increasing concentrations of Antho 50 for 24 h or for 75 μg/mL for the indicated times. Apoptosis in B CLL cells (A, B) and in PBMCs (D) was assessed by flow cytometry using the annexin V-FITC/PI apoptosis assay. Cell viability rate (C) was assessed by cell counting using the trypan blue dye exclusion assay. Equal quantity of genomic DNA was analyzed on a 1% agarose gel. DNA was stained with ethidium bromide and then visualized under UV light (E). The control (Ctr) represents untreated cells harvested at the latest time point. The data are representative of cells from nine CLL patients and four healthy subjects for apoptosis, six CLL patients for cell viability and two for DNA fragmentation. Values are shown as means ± S.E.M. (n = 3); *, *P* < 0.05, **, *P* < 0.01, ***, *P* < 0.001 versus respective control.

To determine whether Antho 50 induces apoptosis in CLL cells, the detection of phosphatidylserine externalization by flow cytometry using annexin V FITC/PI assay kit was performed. As indicated in Fig. 1A, a concentration-dependent increase in annexin V positive cells was observed in Antho 50-treated cells for 24 h and this effect reached significance at concentrations greater than 25 μg/mL of Antho 50. The percentage of annexin V positive cells reached approximately 75% at 75 μg/mL. Incubation of cells with 75 μg/mL of Antho 50 induced a time-dependent increase in annexin V positive cells with a significant effect observed already at 1 h (Fig. 1B) and which was associated with a reduction in cell viability (Fig. 1C). To determine the selectivity of Antho 50, PBMCs from five healthy adult donors were incubated with Antho 50 for 24 h (Fig. 1D). Although Antho 50 at a concentration of 25 μg/mL significantly induced apoptosis in CLL cells by about 50% (Fig. 1A), no such effect was observed in PBMCs (Fig. 1D). However, increasing the concentration of Antho 50 to 75 μg/mL induced a slight but significant apoptosis in PBMCs by about 36% (Fig. 1D). These data indicate that Antho 50 is targeting predominantly neoplastic B cells relative to PBMCs.

DNA fragmentation is a hallmark and also one of the later stages of apoptosis. To confirm the apoptotic mechanism induced by Antho 50, DNA fragmentation analysis was conducted in cells of two CLL patients. The intensity of the genomic DNA smears of the Antho 50-treated CLL cells of both patients increased in a time-dependent manner (Fig. 1E). Altogether, these findings indicate that Antho 50 selectively induces DNA damage-related apoptosis in B CLL cells.

### Delphinidin glycosides induce apoptosis in B CLL cells

The chemical analysis of Antho 50 bilberry extract indicated a polyphenol rich composition (513.20 ± 16.20 mg GAE/g) with a major content of anthocyanins (450.31 ± 5.70 mg/g). Talavéra et al. previously identified 15 major glycoside derivatives including those of delphinidin, cyanidin, petunidin, peonidin and malvidin in Antho 50^13^. In order to determine the proportion of the different anthocyanidins, an UPLC-PDA analysis of the Antho 50 hydrolyzed extract was performed. The Antho 50 content of delphinidin was 102.62 ± 4.87 mg/g, cyanidin 91.05 ± 3.95 mg/g, petunidin 75.99 ± 3.93 mg/g, pelargonidin 1.32 ± 0.05 mg/g, peonidin 9.51 ± 0.44 mg/g and malvidin 84.31 ± 4.73 mg/g. Furthermore, an UPLC-PDA was performed to quantify anthocyanins in their native forms. The Antho 50 content of delphinidin-3*-O-*rutinoside was 89.17 ± 1.67 μg/mg, delphinidin-3*-O-*glucoside 42.60 ± 1.30 μg/mg, cyanidin-3*-O-*glucoside 2.36 ± 0.10 μg/mg, petunidin-3*-O-*glucoside 32.94 ± 0.14 μg/mg and malvidin-3*-O-*glucoside 31.93 ± 0.36 μg/mg.

**Figure 2:**
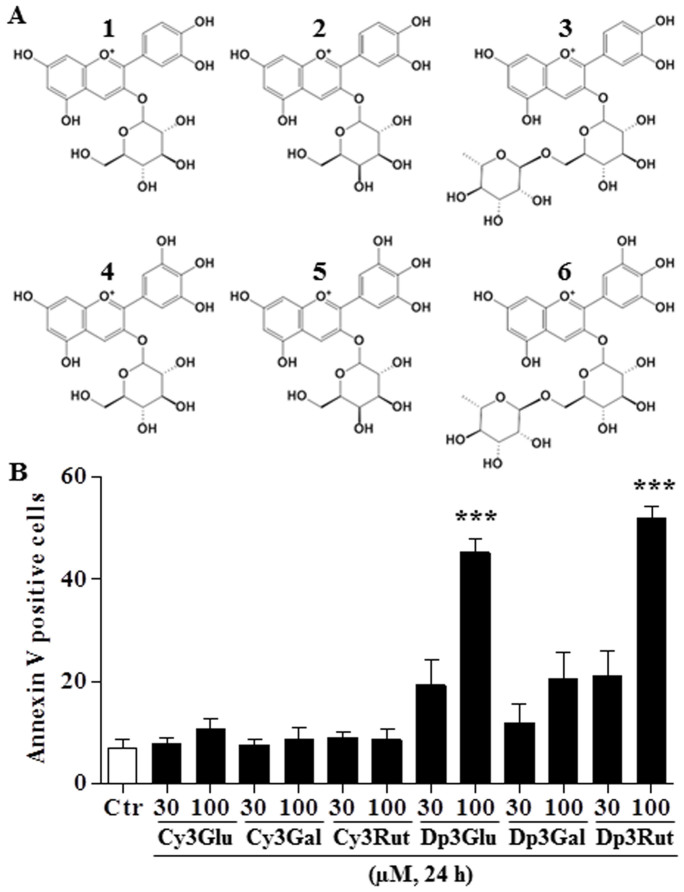
Chemical structures of anthocyanins present in Antho 50 (A): cyanidin-3*-O-*glucoside (1), cyanidin-3*-O-*galactoside (2), cyanidin-3*-O-*rutinoside (3), delphinidin-3*-O-*glucoside (4), delphinidin-3*-O-*galactoside (5) and delphinidin-3*-O-*rutinoside (6). B CLL cells were exposed to 30 or 100 μM of the indicated anthocyanin for 24 h. Then apoptosis rate was determined by flow cytometry using annexin V-FITC/PI assay (B). The control (Ctr) represents untreated cells. The data are representative of cells from four CLL patients. Values are shown as means ± S.E.M. (n = 4); ***, *P* < 0.001 versus control.

In order to determine the pro-apoptotic activity of pure anthocyanins, B CLL cells were incubated for 24 h with 30 or 100 μM of six commercially available anthocyanins: cyanidin-3*-O-*glucoside (**1**), cyanidin-3*-O-*galactoside (**2**), cyanidin-3*-O-*rutinoside (**3**), delphinidin-3*-O-*glucoside (**4**), delphinidin-3*-O-*galactoside (**5**) and delphinidin-3*-O-*rutinoside (**6**) (Fig. 2A). Then, the level of apoptosis was determined by flow cytometry. Treatment of B CLL cells with glucoside and rutinoside delphinidin derivatives increased the percentage of apoptotic cells, whereas delphinidin-3*-O-*galactoside and the cyanidin derivatives had only minor effects (Fig. 2B).

### Antho 50 induces an early caspase 3 activation and UHRF1 down-regulation in B CLL cells independently of the status of tumor suppressor genes *p53* and *p73*

**Figure 3:**
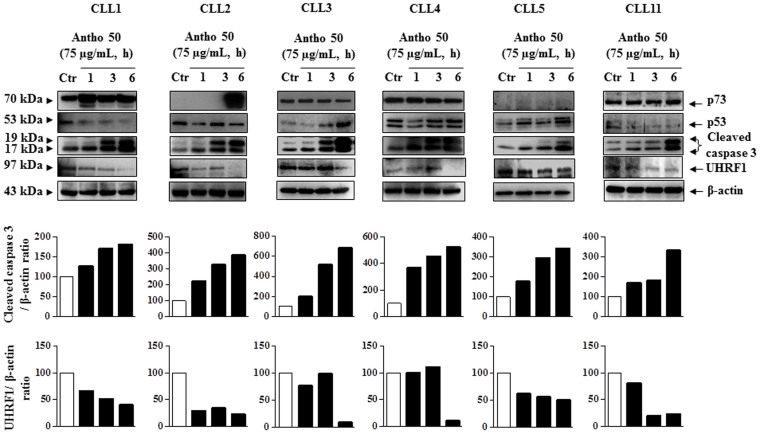
Antho 50 induces caspase 3 activation and UHRF1 down-regulation independently of p53 and p73. B CLL cells were incubated with Antho 50 at 75 μg/mL for the indicated times and thereafter the expression of the p53, p73, cleaved caspase 3 and UHRF1 was studied using Western blot. The control (Ctr) represents untreated cells harvested at 6 h. The data are representative of cells from six CLL patients. Cleaved caspase 3 and UHRF1 expression levels were analyzed by densitometry and represented as percentage compared with control.

Activation of caspase-dependent cascade leading to apoptosis has been involved in polyphenolic extracts-mediated cell death in cancer cells including leukaemia^17^,^18^,^19^,^20^. We therefore determined the involvement of activated caspase 3, one of the main executors of apoptosis in the pro-apoptotic effect of Antho 50 in CLL cells (Fig. 3). Exposure of cells to 75 μg/mL of Antho 50 induced a time-dependent caspase 3 activation (Fig. 3). A slight increased expression of activated caspase 3 was observed already at one h and thereafter the signal increased progressively at least until 6 h (Fig. 3).

UHRF1 (Ubiquitin-like containing PHD and ring finger domains 1), a potent oncogene overexpressed in many human cancer cells, has been shown to play an important role in the epigenetic silencing of various tumor suppressor genes^21^,^22^,^23^. Several reports have indicated that UHRF1 overexpression promotes proliferation of cancer cells by inhibiting apoptosis suggesting that this oncogene is a new therapy target for cancer cells, including leukaemia^23^,^24^,^25^,^26^. As shown in Fig. 3, treatment of CLL cells with Antho 50 induced a decrease in UHRF1 expression accompanied by progressive activation of caspase 3 providing further evidence for the pro-apoptotic properties of Antho 50. These results suggest that Antho 50-induced apoptosis is linked to a rapid caspase 3 activation and UHRF1 down-regulation in CLL cells. To characterize the Antho 50-induced pro-apoptotic signaling pathway leading to caspase 3 activation, the levels of the tumor suppressor proteins p53 and p73 were determined. As shown in Fig. 3, the levels of p53 remained either unchanged or decreased in most CLL cells samples (CLL1, CLL2, CLL4, and CLL11). In the samples isolated from patients 3 and 5, an increase in p53 expression was observed at 6 h and caspase 3 activation at 3 h (Fig. 3). The level of p73, a pro-apoptotic member of the p53 family was undetectable or unchanged in 4 out of 6 CLL samples (Fig. 3). In the case of CLL 2, a pronounced increase in the level of p73 was observed at 6 h and caspase 3 activation at 1 h, and in the case of CLL 1, an increased level of p73 was observed in parallel with caspase 3 activation (Fig. 3). Taken together, these results suggest that the pro-apoptotic cellular response of CLL cells to Antho 50 involves caspase 3 activation and UHRF1 down-regulation predominantly via p53- and p73-independent pathways.

### Antho 50 induces Bcl-2 down-regulation associated with Bad (Bcl-2-associated death promoter) dephosphorylation

**Figure 4:**
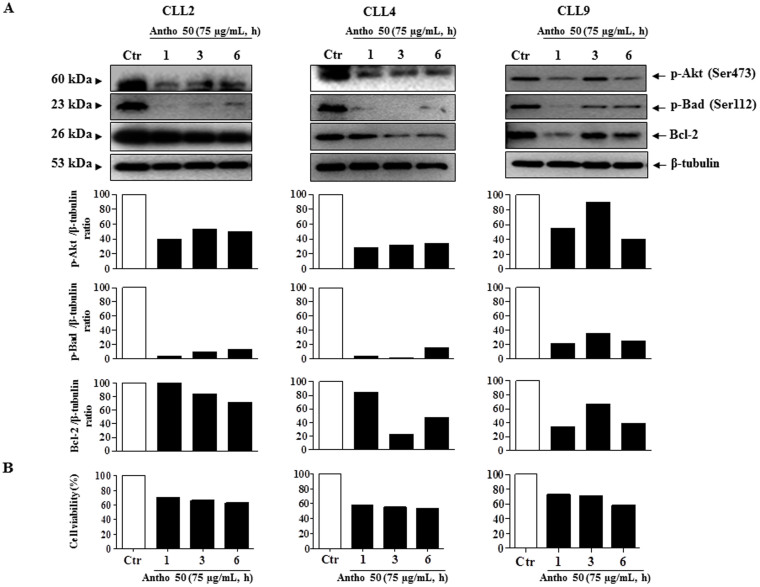
Antho 50 induces dephosphorylation of Akt at Ser473, Bad at Ser112 and down-regulation of Bcl-2 in B CLL cells. Cells were incubated with Antho 50 at 75 μg/mL for the indicated times. The expression of the p-Akt, p-Bad and Bcl-2 was studied by Western blot (A, upper panel) and their expression levels were analyzed by densitometry and represented as percentage compared with control (A, lower panel). Cell viability was assessed by cell counting using the trypan blue dye exclusion assay (B). The control (Ctr) represents untreated cells harvested at 6 h. The data are representative of cells from three CLL patients.

The Bcl-2 family plays a key regulatory role in cellular responses to treatment via its pro- and anti-apoptotic properties^27^. The anti-apoptotic protein Bcl-2 is overexpressed in several hematological malignancies including CLL and this overexpression is considered primarily responsible for defective apoptosis in CLL^28^. We therefore evaluated the effect of Antho 50 treatment on the expression of two major members of the Bcl-2 family, Bcl-2 and p-Bad in cells from 3 CLL patients. As shown in Fig. 4A, a reduced Bcl-2 level was observed in CLL cells as a function of the treatment time with Antho 50. The down-regulation of Bcl-2 was accompanied by a reduction of cell viability starting at 1 h (Fig. 4B).

Since the inactivation of p-Bad via its dephosphorylation induces Bcl-2 down-regulation leading to apoptosis^29^, the state of p-Bad was examined in response to Antho 50 treatment. Antho 50 treatment induced an early pronounced dephosphorylation of Bad starting at 1 h, which was followed with the down-regulation of Bcl-2 (Fig. 4A). Since p-Akt can phosphorylate Bad at Ser112 and Ser136 promoting survival^30^, the potential of Antho 50 to inhibit the constitutive phosphorylation of Akt in CLL cells was examined. As indicated in Fig. 4A Antho 50 caused dephosphorylation of Akt at Ser473 within 1 h. These findings suggest that Antho 50 treatment causes dephosphorylation of Akt at Ser473 leading to the subsequent dephosphorylation of Bad, and ultimately the down-regulation of Bcl-2 producing activation of the caspase 3-related apoptotic pathway.

### Antho 50 induces apoptosis in CLL cells through generation of ROS

**Figure 5:**
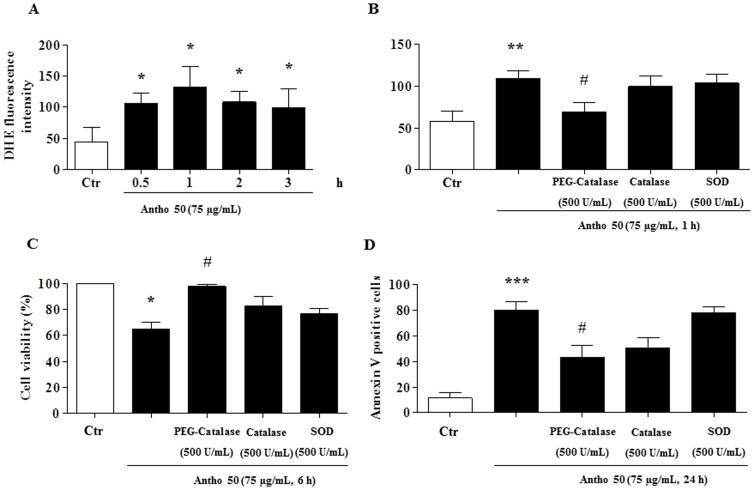
Antho 50 decreases cell viability and triggers apoptosis in B CLL cells through generation of ROS. Cells were exposed to Antho 50 (75 μg/mL) for different times (A) or to various inhibitors of ROS (B) for 30 min before the addition of Antho 50 (75 μg/mL) for 1 h. The formation of ROS was assessed by flow cytometry after incubation with the redox-sensitive fluorescent probe DHE. Cells were incubated with various inhibitors of ROS for 30 min before the addition of Antho 50 (75 μg/mL) for 6 h for cell viability analysis (C) or for 24 h before the determination of apoptosis (D). The control (Ctr) represents untreated cells harvested at 6 h. The data are representative of cells from three CLL patients for ROS analysis, three CLL patients for cell viability, and four CLL patients for apoptosis analysis.

Several *in vitro* and preclinical studies have shown that anti-cancer drugs and natural products including polyphenols induce pro-apoptotic effects in hematological cancer cells including CLL cells through the generation of ROS^18^,^19^,^31^,^32^. Therefore experiments were performed to determine whether Antho 50 stimulates the formation of ROS in B CLL cells and, if so, it's role in apoptosis. As indicated in Fig. 5A, Antho 50 caused within 30 min a significant increase in the formation of ROS in CLL cells as assessed using the redox-sensitive probe DHE. To determine whether Antho 50-increased ROS formation is an intracellular or an extracellular event, the effect of various antioxidants was examined. Fig. 5B indicates that a marked reduction of the formation of ROS was observed when Antho 50-treated CLL cells were pre-incubated with the membrane-permeant analog of catalase (PEG-catalase). In contrast neither native SOD nor catalase had such an effect (Fig. 5B). Exposure of cells to the intracellular antioxidant PEG-catalase significantly prevented the Antho 50-induced reduction in cell viability (Fig. 5C) and apoptosis (Fig. 5D) whereas native SOD and catalase had only slight effects. Thus, these observations indicate that Antho 50 predominantly stimulates the intracellular formation of ROS and that this effect is an upstream event in the Antho 50-induced apoptosis.

### Antho 50 affects apoptosis-regulating proteins in CLL cells through a redox-sensitive mechanism

**Figure 6:**
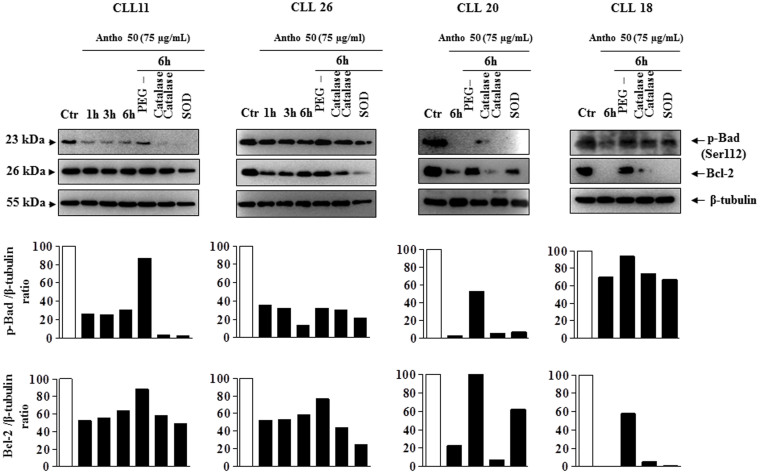
Antho 50 affects p-Bad and Bcl-2 proteins through a ROS-dependent mechanism. B CLL cells were exposed to either PEG-catalase (500 U/mL), catalase (500 U/mL), or SOD (500 U/mL) for 30 min before the addition of Antho 50 (75 μg/mL) for the indicated times. The expression of p-Bad and Bcl-2 was studied using Western blot and their expression levels were analyzed by densitometry and represented as percentage compared with control (Ctr). The control represents untreated cells harvested at the latest time point. The data are representative of four CLL patients.

**Figure 7:**
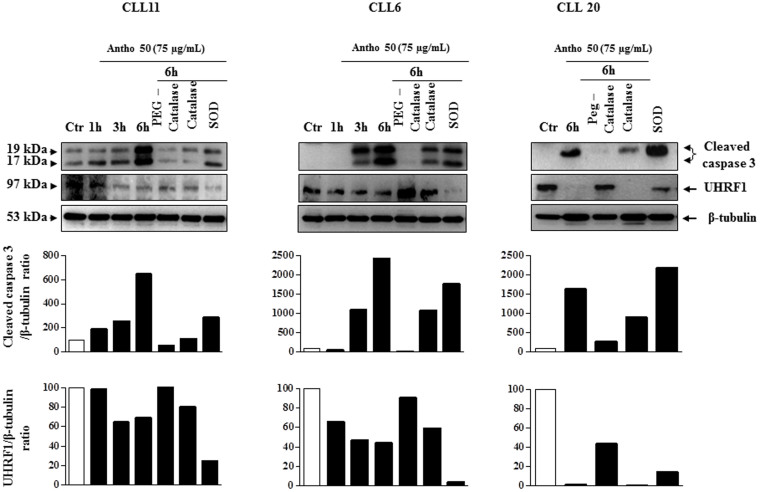
Antho 50 affects cleaved caspase 3 and UHRF1 proteins through a ROS-dependent mechanism. B CLL cells were exposed to either PEG-catalase (500 U/mL), catalase (500 U/mL), or SOD (500 U/mL) for 30 min before the addition of Antho 50 (75 μg/mL) for the indicated times. The expression of cleaved caspase 3 and UHRF1 was studied using Western blot and their expression levels were analyzed by densitometry and represented as percentage compared with control (Ctr). The control represents untreated cells harvested at 6 h. The data are representative of three CLL patients.

To determine whether the intracellular formation of ROS is a key event in the Bad/Bcl-2 deregulation leading to activation of the caspase 3-related pro-apoptotic signaling pathway in response to Antho 50, the effect of various antioxidants were tested. Exposure of cells isolated from patient 11, 26, 20 and 18 with PEG-catalase markedly reduced the Antho 50-induced dephosphorylation of Bad and down-regulation of Bcl-2 at 6 h (Fig. 6). Native SOD and catalase affected only slightly or had no such effect on p-Bad and Bcl-2 (Fig. 6). The Antho 50-induced activation of caspase 3 was inhibited in CLL cells CLL11, 6 and 20 (Fig. 7) and CLL9 and 10 (data not shown) in the presence of PEG-catalase as well as the down-regulation of UHRF1 in CLL11, 6 and 20 (Fig. 7). In contrast, native SOD and catalase affected only slightly or not at all both signals (Fig. 7). Altogether, these findings indicate that Antho 50 triggers apoptosis in CLL cells through a redox-sensitive activation of the caspase 3-related pro-apoptotic pathway possibly through Bad dephosphorylation and Bcl-2 down-regulation.

## Discussion

Among hematological cancers, CLL is considered as a characteristic example of a neoplasia caused by the failure of apoptosis^4^,^33^. Because of the important chemotherapy resistance and drug toxicity observed in treatment of this malignancy, there is a need for development of new therapeutic approaches. The Bcl-2 family proteins have a central role in CLL cell survival and chemotherapy resistance^1^, making Bcl-2 inhibition as a potent target to induce apoptosis in CLL cells. The present study indicates that a polyphenol-rich extract (Antho 50) decreased cell viability and induced concentration- and time-dependent apoptosis in cells isolated from CLL patients. Interestingly, Antho 50 had no or only a weak effect in PBMC isolated from healthy subjects. The present study also sheds light onto the mechanism underlying the Antho 50-induced apoptosis in B CLL cells. Pharmacological inhibition of ROS formation indicated the involvement of a redox-sensitive event in the caspase 3-related pathway in Antho 50-induced apoptosis of B CLL cells. The present study is in good agreement with recent reports indicating that anthocyanins induce mainly a caspase 3-dependent apoptosis in cell lines derived from colorectal cancer, and monocytic and promyelocytic hematological malignancies^30^,^34^,^35^,^36^,^37^. The present findings extend these observations to the most frequent hematological malignancy, CLL, as indicated by the observations with cells from 30 patients.

The present findings provide evidence for a molecular action of Antho 50 in CLL involving activation of the caspase 3-related apoptotic pathway, as a result of down-regulation of Bcl-2 subsequent to Bad dephosphorylation. They further indicate that a rapid formation of ROS is involved in Antho 50-induced apoptosis. It is well-known that activation of Akt promotes cell survival by targeting several proteins involved in the regulation of apoptosis such as the pro-apoptotic Bcl-2 family member Bad^30^. The present findings indicate that Antho 50-mediated rapid dephosphorylation of Akt is associated with dephosphorylation of Bad protein at Ser112 and reduced cell viability. It has been shown that the dephosphorylation of Bad promotes cell death by interacting with the anti-apoptotic protein Bcl-2 causing its down-regulation, which allows the activation of the mitochondria-mediated pro-apoptotic pathway leading to caspase 3 activation and cell death^38^. Antho 50 rapidly induced Bad dephosphorylation in parallel with Bcl-2 down-regulation, caspase 3 activation and apoptosis. Previous reports have also indicated that polyphenolic compounds such as resveratrol, quercetin, curcumin, carnosic acid, and silibinin induced apoptosis via caspase 3 activation in leukaemia cells^17^,^39^.

In addition, Antho 50 caused down-regulation of the epigenetic integrator UHRF1, an anti-apoptotic protein which is overexpressed in many human cancer cells and plays an important role in the epigenetic silencing of various tumor suppressor genes including *p16^INK4A^*, hMLH1 and RB1^21^,^22^,^23^,^40^. The histone deacetylase (HDAC) inhibitor valproate has been shown to act synergistically with fludarabine and cladribine, two clinically used anticancer drugs in the treatment of CLL cells^41^. Several reports have indicated that the anti-cancer drugs-induced inhibition of UHRF1 activity and/or expression might prevent the action of two of its preferred partners, namely HDAC1 (histone deacetylase 1) and DNMT1 (DNA methyltransferases), leading to re-expression of several tumor suppressor genes and thus allowing cancer cells to undergo apoptosis^21^,^40^,^42^. In agreement with these observations, Antho 50 induced UHRF1 down-regulation, such a response may lead to reduced HDAC1 activity and as a consequence an increased apoptosis in CLL cells. Altogether, these findings suggest that Antho 50 induces a combined effect to kill CLL cells through caspase 3 activation and inhibition of UHRF1-regulated expression of several proteins involved in the repression of tumor suppressor genes.

Several reports have shown that polyphenolic compounds can increase ROS levels in human cancer cells, including leukemia and that this response is involved in the pro-apoptotic effect of red wine polyphenols, *Aronia melanocarpa* polyphenols and EGCG through UHRF1 down-regulation and caspase 3 activation in leukemia cells^18^,^19^. Similarly, in the present study Antho 50 treatment of CLL cells rapidly induced an increased formation of ROS causing a reduction in cell viability and induction of apoptosis. The intracellular antioxidant PEG-catalase prevented the Antho 50-induced formation of ROS, reduction of cell viability and induction of apoptosis indicating a determinant role of ROS. In addition, the antioxidant PEG-catalase inhibited also the Antho 50-induced Bad dephosphorylation, Bcl-2 and UHRF1 down-regulation and caspase 3 activation providing further evidence that ROS play a key role in the Antho 50-induced apoptosis in CLL cells.

Polyphenols, particularly anthocyanins, have been reported to mediate the pro-apoptotic properties of different berries in various types of cancer cells, including those from colon tumors and leukemia^19^,^43^,^44^. Our findings have identified the glucoside and rutinoside derivatives of delphinidin as active components of Antho 50 involved in the induction of apoptosis. Katsube et al. also observed that delphinidin strongly inhibited the growth of HL60 human promyelocytic leukemia cells whereas cyanidin had little effect^45^. Altogether, these observations suggest that a hydroxyl group on position 5′ of the B ring is a key structural characteristic involved in B CLL cytotoxicity. Since Antho 50 is a complex mixture of phytochemicals, and the pro-apoptotic effect of the extract is superior to that attributable to two major anthocyanins (delphinidin-3*-O-*glucoside and delphinidin-3*-O-*rutinoside) of Antho 50, anthocyanins and possibly also other polyphenols, as flavonols and chlorogenic acid, might act in synergy to induce apoptosis in B CLL cells^46^,^47^.

**Figure 8:**
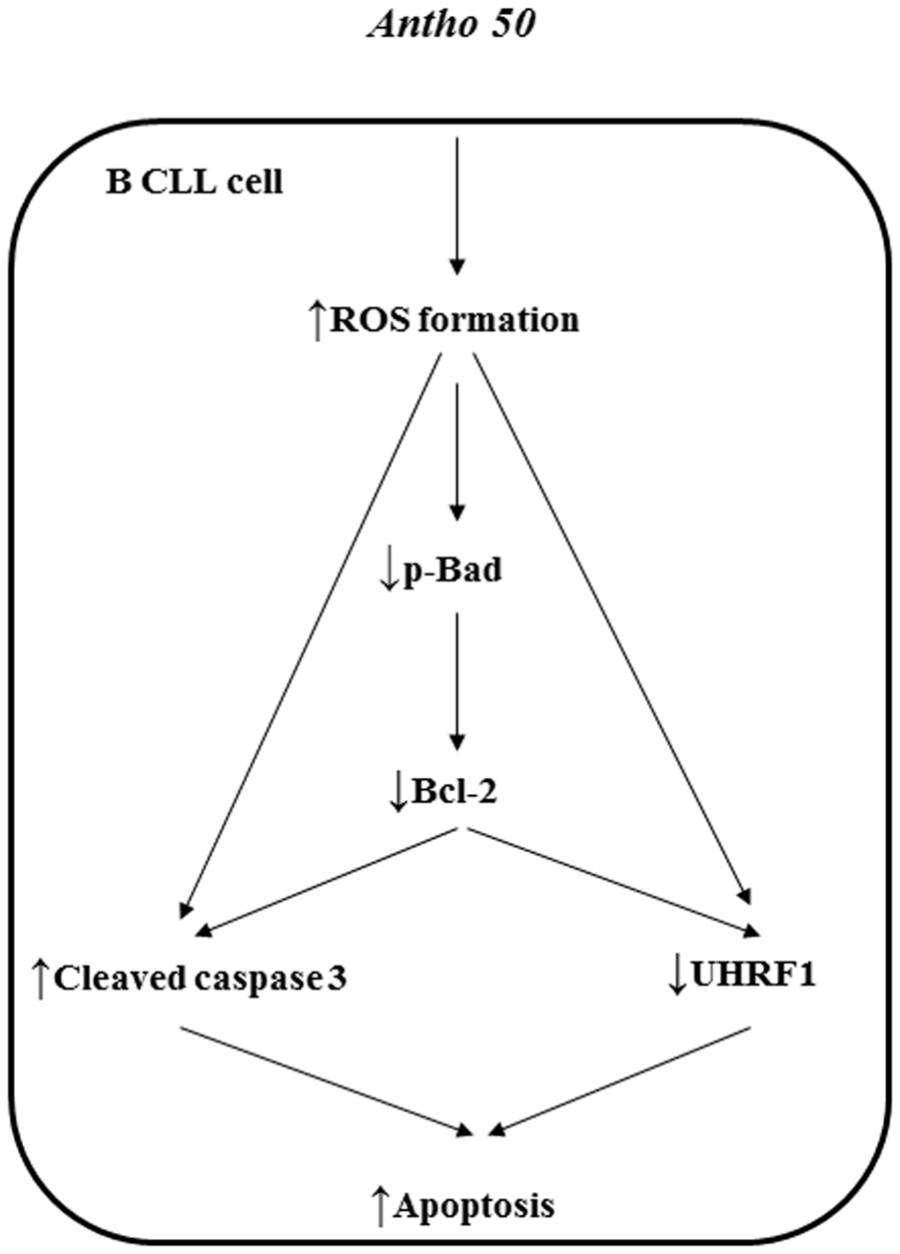
Schematic summarizing the pro-apoptotic signaling cascade in B CLL cells induced by Antho 50.

In conclusion, the present study highlights the potential of Antho 50 to induce a redox-sensitive apoptosis in CLL cells with little effect on healthy PBMC. Delphinidin-3-*O*-glucoside and delphinidin-3-*O*-rutinoside were identified as active anthocyanins. It further suggests that Bcl-2 which is known to protect CLL cells from apoptosis, is a major target for Antho 50 and that its degradation via Bad dephosphorylation leads to caspase 3 activation (Fig. 8).

## Author Contributions

M.A., A.J.L.G., I.D., A.L., C.K., J.P. and S.R. carried out the experiments. V.S.K., R.H. and M.A. performed the study design, data acquisition and analysis and wrote the manuscript. L.M.F. and L.M. contributed to interpretation of data and study coordination.

## Figures and Tables

**Table 1:** Clinical characteristics of the CLL patients

Patient no.	Sex	Age (y)	Absolute lymphocytosis, ×10^3^/μL	Prior treatment status
1	F	60	54.9	Treated
2	M	81	84.4	Untreated
3	M	77	43.8	Untreated
4	M	70	190.2	Treated
5	M	76	80.0	Treated
6	M	64	101.2	Untreated
7	F	73	66.1	Treated
8	F	53	103.6	Untreated
9	M	68	6.9	Treated
10	M	70	45.5	Untreated
11	M	77	56.9	Untreated
12	M	75	11.6	Untreated
13	M	71	58.0	Untreated
14	F	71	4.2	Untreated
15	M	82	11.9	Untreated
16	M	73	14.2	Untreated
17	M	78	25.2	Untreated
18	F	67	18.9	Untreated
19	F	43	7.4	Untreated
20	M	61	17.8	Untreated
21	F	66	35.9	Treated
22	M	61	32.5	Untreated
23	M	81	9.5	Untreated
24	F	83	182.6	Untreated
25	M	56	68.4	Untreated
26	M	68	31.8	Treated
27	M	57	61.9	Untreated
28	M	79	30.3	Untreated
29	M	67	21.5	Untreated
30	F	66	122.5	Untreated
